# Fostering Healthcare Innovation: A Mixed-Methods Study of an Impact Entrepreneurship Course for Nurse Practitioner Students

**DOI:** 10.3390/nursrep15110397

**Published:** 2025-11-12

**Authors:** Zvika Orr, Beth G. Zalcman, Ronit Pinchas-Mizrachi, Anat Romem

**Affiliations:** 1 Selma Jelinek School of Nursing, Jerusalem College of Technology, 11 Beit Hadfus Street, Jerusalem 9548311, Israel; beth.zalcman@biu.ac.il (B.G.Z.); pinchasm@g.jct.ac.il (R.P.-M.); 2 CERMES3—Centre for Research on Medicine, Science, Health, Mental Health, and Society, CNRS—INSERM—EHESS—Université Paris Cité, 7 Guy Môquet Street, 94801 Villejuif, France; 3 The Henrietta Szold Hadassah—Hebrew University School of Nursing, Ein Kerem Campus, Kalman Y. Man Street, Jerusalem 91120, Israel; romem@g.jct.ac.il

**Keywords:** impact entrepreneurship, innovation, curriculum, nurse practitioners, nursing education

## Abstract

**Background/Objectives:** Despite its benefits for both nurses and patients, entrepreneurship among nurses remains uncommon. Specifically, impact entrepreneurship has rarely been applied in nursing. Impact entrepreneurship promotes solutions to health, social, and environmental challenges using business models committed to measurable social impact and return on investment. Beginning in 2020, an impact entrepreneurship course was introduced as a mandatory component of the master’s and nurse practitioner programs in geriatrics and palliative care at an Israeli college. This article examines the perceptions of the nurses who completed the course and explores how the course affected them and their professional practice. **Methods**: This mixed-methods study employed a convergent design and included self-administered questionnaires sent to all graduates of two cohorts, along with a qualitative thematic analysis of students’ reflective essays written during the course. **Results**: Students reported that the course empowered them to be innovators and contributed to their professional and personal development. They also explained that studying impact entrepreneurship could increase their earning potential and enable them to make a substantial difference for patients. Throughout the course, participants shifted their view of entrepreneurship and realized that, given nurses’ closeness with patients, they can and should be entrepreneurs. Before the course, students were reluctant to engage in entrepreneurship; afterward, students demonstrated marked interest in pursuing entrepreneurial pathways. **Conclusions**: Exposure to impact entrepreneurship can help nurses identify and implement creative and cost-effective solutions to workplace challenges. The professional characteristics of nurses position them as potential leaders of impact entrepreneurship in healthcare. Educational institutions worldwide should incorporate the subject of impact entrepreneurship into curricula and practice to realize that potential.

## 1. Introduction

In recent years, there has been growing recognition of the contribution of the individual innovation of nurses and nursing students [1,2,3,4]. Nurses’ innovative practice often includes entrepreneurial initiatives, which are increasingly regarded by healthcare organizations as facilitating dynamism, creativity, and efficiency in the sector, improving patient outcomes, and reducing costs [5,6,7,8]. Studies have shown that entrepreneurial leadership has significant effects on nurses’ proactive work behavior and career adaptability [9]. However, little is known on how innovation and entrepreneurship programs in medical and nursing schools construct the perceptions, knowledge, skills, and professional practices of learners, as well as on the strategies that can facilitate learners’ adjustment to complex professional roles as entrepreneurs [5,10,11,12,13,14,15]. Specifically, impact entrepreneurship programs for nurses have rarely been implemented and studied. Impact entrepreneurship is the promotion of sustainable solutions that address health, social, and environmental challenges using a business model committed to both measurable social impact and return on investment [16]. This mixed-methods article aims to contribute to bridging these gaps by analyzing how a unique course on impact entrepreneurship was experienced by nurse practitioner students who completed it and how the course affected the nurses, their perceptions, and their professional practice.

### 1.1. Entrepreneurship Among Nurses

Nursing entrepreneurship enables nurses to be initiators of change and employ their passion to improve healthcare using innovative approaches [1,17]. A nurse entrepreneur is often a business owner who provides nursing services, in whatever capacity; this can be an independent clinical practice, a consultancy, or another healthcare-related business. Sometimes, a nurse employed within a healthcare system will be the one to develop a new service or product for patients or other healthcare providers. These nurses are intrapreneurs, and they are driven by the specific needs of the patients that they treat in their facility [1,8,17].

Both nurse entrepreneurs and intrapreneurs can fill gaps in the healthcare system, by recognizing needs and challenges facing both patients and providers and offering solutions in the form of a new product or service [18]. Nurse entrepreneurs, whose ideas become viable businesses, have greatly contributed to advances in healthcare, including more effective service models, better patient outcomes, and better patient experience [19,20]. Entrepreneurship also encourages nurses to be creative and help redefine the parameters of the role [21].

Entering the world of entrepreneurship can be empowering for nurses [15,22]. Psychological empowerment has been defined as “a cognitive, subjective and motivational process by which individuals perceive themselves as effective and competent for carrying out tasks … Moreover, the tasks themselves are deemed relevant and meaningful, and individuals feel they have freedom of choice in relation to them” [23]. Accordingly, nurse entrepreneurs and intrapreneurs tend to experience greater effectiveness, competence, and autonomy [1,15,19,22].

However, despite the potential benefits to nurse entrepreneurs, entrepreneurship among nurses is not a common phenomenon. The reported prevalence of nursing entrepreneurs is low, approximately 0.5–1% of all nurses [21,24]. Furthermore, entrepreneurship-related education among nurses is sparse [24]. They lack a background in business administration and do not have the business-related networks [20]. Nurse entrepreneurs must also contend with how outsiders view the nursing profession in its traditional sense [25]. This is true of nurse intrapreneurs as well, though there are places of employment that may offer more support and encourage entrepreneurship among nurses. Furthermore, there is a lower risk involved in terms of employment status, as nurse intrapreneurs still work within a healthcare system [26]. The capabilities of nurses to engage in entrepreneurship are also affected by their personal characteristics, such as their approach towards risk, creativity, and organizational skills, as well as their occupational background, including their knowledge, training, and experience [27].

Given the important contribution of entrepreneurship in nursing, this subject is underdeveloped in nursing education and other healthcare education programs [10,11,28]. Existing programs have highlighted the importance of enhancing nurses’ understanding of the topic’s relevance to their practice and translating business terminology into nursing-specific language, thereby addressing the common initial perception that entrepreneurship is irrelevant to nursing [14]. Research has shown that students’ innovation is fostered through teamwork, interprofessional collaboration, mentor support, and engagement with external partners [13]. Educational programs empowered nursing students by reframing nurses as potential entrepreneurial actors, enabling them to identify problems, define actionable solutions, and take initial steps toward testing those solutions [15]. Entrepreneurship education enabled nursing students to develop professional identity values, including critical thinking, leadership, and political awareness, allowing them to challenge the status quo [10]. However, while recognized as necessary in order to encourage entrepreneurship and innovation by nurses, there are few courses offered to nurses on this topic [29,30]. Scholars and practitioners emphasized the importance of conducting research in diverse contexts to advance understanding of entrepreneurial education for nursing students [12].

### 1.2. Impact Entrepreneurship

Impact entrepreneurship is a business enterprise that is ethical and transparent, and makes a measurable impact in the social, environmental, and health issues it seeks to address. It promotes solutions that overcome grand environmental, social, and health challenges, including global challenges, using a business model committed to both measurable social return and return on investment. Impact entrepreneurs are not looking to create a product that will dominate their market but, rather, develop sustainable solutions, particularly for issues considered to be of the public domain [16]. Impact entrepreneurship evolves through continuous collaboration and negotiation among entrepreneurs, investors, executives, and mentors in the everyday practices that Dumont calls the “impact work” [31].

The concept of impact entrepreneurship is related to social entrepreneurship, which is more prevalent in nursing. Social entrepreneurship applies market mechanisms to achieve financial stability, foster community self-sufficiency, and create ventures aimed at addressing pressing social challenges [28]. Social entrepreneurship involves creating social value by recognizing opportunities, applying innovation, taking above-average risks, and resourcefully pursuing solutions despite limited resources [32].

The intersection between nurse entrepreneurs and impact entrepreneurship is a natural meeting point. Many times, nurse entrepreneurs are motivated to develop their enterprises in order to improve patient health outcomes or provide more efficient services, reflecting how nurses drive innovation for the greater good of society [17,20]. With all the current challenges and technological developments that are shaping the modern healthcare system, nurses have an opportunity to offer solutions, through a unique perspective and close encounter with the healthcare system as well as individual patients [18,27,33]. However, the challenges facing nurses who engage in impact entrepreneurship are similar to nurse entrepreneurs. These include a narrow view of nursing by other players in the healthcare system, feelings of excessive regulatory practices, and lack of business acumen [18,30]. Additionally, despite the natural meeting point between nursing and impact entrepreneurship, the latter is rarely taught during nursing studies, making it difficult for nurses to later contribute to this field [30].

### 1.3. Impact Entrepreneurship for Geriatric Nursing

In Israel and around the world, the older adult population is steadily increasing, due to a longer life expectancy. By 2030, the number of older adults in Israel (aged 65 and older) is expected to comprise over 13% of the total population [34]. Longer life expectancy is accompanied by an increase in morbidity, as well as new social and economic challenges, and health and welfare systems are struggling to cope with this trend [35,36,37].

There is increasing recognition that older adults constitute a significant segment of the market. There is a need for new technologies and services to address the needs of this population, such as frequent use of medical services, assistance in activities of daily living, and well-being. However, there is still a lack of interest on the side of entrepreneurs to engage in gerontechnology. Entrepreneurs require knowledge about various aspects of aging, including the challenges faced by older adults, in order to engage in gerontechnology [38].

Studies have demonstrated the effectiveness of geriatric nurses in clinical care, specifically in improving quality of life and well-being, as well as in economic metrics [39]. Specialist nurses, such as nurse practitioners in geriatrics, can play a key role in impact entrepreneurship and policy-making for older adults. Impact initiatives aim to address significant challenges facing older adults, and geriatric nurses are well-versed in these challenges. Therefore, geriatric nurses are in a unique position to initiate impact projects to provide solutions for these challenges. However, there is very limited literature relating to impact or social entrepreneurship for older adults, and no literature related to impact entrepreneurship by geriatric nurses. The present article helps bridge this gap by examining a course on impact entrepreneurship for older adults, designed specifically for geriatric and palliative care nurses.

### 1.4. The Impact Entrepreneurship Course

Beginning in 2020, an impact entrepreneurship course was incorporated as a mandatory course to nursing students studying in the Master of Science in Nursing (MSN) and Nurse Practitioner (NP) program (MSN + NP) at the Jerusalem College of Technology. All students in this program were experienced nurses with at least two and a half years of professional experience, many with two or even three decades in the field, specializing in either geriatrics or palliative care. The students in the course had previously completed research-related courses and had a solid grasp of evidence-based practice. To date, 187 nurses have completed this course across five cohorts (2020–2025).

The course was conducted over a single semester, with each class lasting three hours. Its goals were:

(1) To promote social, environmental, and health causes, specifically global and local challenges related to aging, through a business model committed to both measurable social outcome and economic profitability (return on investment).

(2) To empower nurse practitioners to engage in entrepreneurship and intrapreneurship and to become agents of change.

(3) To encourage nurses to emerge as leaders in impact entrepreneurship in Israel, thereby establishing this field as an accepted and thriving area within nursing.

The first and last authors initiated the course, and all authors taught it intermittently over different years (collectively amounting to five years of instruction). We designed the curriculum to combine theory, practical knowledge of entrepreneurship and innovation, and social action. The course was structured as an interactive, multidisciplinary program and included guest lectures from experts in innovation and business, who, together with the authors, mentored the students throughout the semester. The students learned the basic concepts of the field of impact entrepreneurship, financial instruments, the components of impact investments and investment strategies, and methods for measuring social and financial return on investment. They learned about the multiple actors involved in impact entrepreneurship [31], the interconnections between the economy, society, medicine, and nursing, and the socio-environmental responsibilities of healthcare providers and institutions. Thus, the course complemented other socially engaged programs at the college that promoted social responsibility [40,41]. The course also addressed the primary challenges that impact entrepreneurship may face.

Additionally, during the course, students visited organizations that exemplified impact entrepreneurship. The specific contents of the field trips have changed over the years. Usually, the first field trip was to an organization that works with and for pediatric oncology patients, including the provision of technology to help them preserve quality of life. The second field trip was to see community-based initiatives, such as a non-profit organization that supports active older adults in Jerusalem by providing them with creative outlets and platforms through which to sell their work.

During the course, students applied the theories and knowledge they learned about impact entrepreneurship for older adults to final projects. They usually worked in groups of two to four students, and some preferred to work alone, to initiate, plan, develop, and present their projects. Some projects were new services, such as a hospice in southern Israel, a strategy for better daily medication management, a therapeutic garden for older adults, and one-stop accessible hearing services for seniors, including tests, aids, and care. Other projects were new innovative products, including a specialized sleeve to prevent hot water urns from falling over, home-based rehabilitation for older adults after stroke using a virtual therapeutic space, a spelt sock to prevent pressure ulcers on ankles, and special bracelets with advance directives. The students’ projects were primarily formative assessments, designed to support learning, develop skills in impact entrepreneurship, foster professional growth, and facilitate the practical application of course concepts.

The teaching staff identified outstanding, novel but realistic projects with a high potential to be successfully implemented in the healthcare system soon. The students who created these projects were offered a scholarship and ongoing multi-professional guidance and assistance (e.g., in business and product design) to further develop and implement their projects. Twelve projects were selected to receive this support from the Edmond de Rothschild Foundation, which supported the course. Many of these projects were successfully implemented in the healthcare system, while other students currently continue to develop and implement their innovative projects. One example of a project successfully implemented by three students is an intervention program for the care of homebound individuals, led by geriatric nurse practitioners. The students obtained funding for the project and recruited two nurse practitioners who currently operate the program in two regions of the country. Furthermore, six projects were presented as posters at a national conference in Jerusalem in 2025, and other students presented their projects in various professional meetings. However, the data presented in this article do not reflect this stage of the program but the stages in which all the students in the course participated.

This article examines how the impact entrepreneurship course was perceived by the nurse practitioner students who completed it, and how it influenced them and their professional practice. This includes, for instance, the course’s contribution to students’ knowledge, skills, and professional and personal development; their views on nurses as entrepreneurs and innovators; and the likelihood that they will continue to engage in entrepreneurship after the course. It is noteworthy that, although the distinction between entrepreneurship and intrapreneurship was demonstrated in the course, this article treats impact intrapreneurship as part of the broader and more established concept of impact entrepreneurship to maintain analytical focus.

## 2. Materials and Methods

This mixed-methods study employed a convergent design and included a cross-sectional, web-based survey conducted among course graduates, as well as a thematic analysis of student reflections written throughout the duration of the course. Survey and essay data were collected across partially overlapping cohorts and analyzed separately, and then integrated to provide complementary insights into students’ perceptions [42,43].

### 2.1. The Quantitative Component

#### 2.1.1. Study Design and Population

The quantitative component of the study employed a cross-sectional design using a census sampling approach within two selected cohorts of the course. All students who had taken the course in these two cohorts were eligible to participate. There were no exclusion criteria. The survey was therefore sent to all 82 eligible students, representing 43.9% of all course graduates across five cohorts. No a priori sample size calculation was conducted, as the survey targeted the entire population of eligible students within these cohorts. A total of 46 students responded (56.1% response rate).

#### 2.1.2. Materials

Data were collected through distributed questionnaires, which were self-administered. Three months after the end of the course (in 2022 and 2023), the students were sent a survey via e-mail. This period was selected in order to see the course mediocre effect. The authors designed the Impact NP Questionnaire. A panel of five impact entrepreneurship experts validated the questionnaire. They examined whether the questionnaire items could adequately measure the intended concepts, and whether the items were sufficient to evaluate the domain of interest. Cronbach’s alpha on the Impact NP Questionnaire was 0.870 in the present sample.

The questionnaire included sociodemographic and occupational characteristics—specifically age, sex, marital status, religious observance, work setting, level of nursing education, and years of nursing experience. Seven variables captured the respondents’ perspectives on impact entrepreneurship and their impact project: whether they had previous experience in entrepreneurship or in the field of impact (yes/no); whether their impact project was based on their personal experience at work (yes/no); whether it was based on their personal experience as a patient or relative of patients in the healthcare system (yes/no); the extent to which entrepreneurship could be a job change for the student (on a 6-point Likert scale: 1 = not at all, 6 = yes, absolutely); whether they believed the project they proposed was feasible (yes/no/maybe, but not now); whether they had undertaken any activities to promote the implementation of their proposed project (yes/no); and whether they thought there were gender gaps in entrepreneurship and innovation (yes/no/not sure). These variables were measured using items developed for this study.

Quantitative data were analyzed using Statistical Package for the Social Sciences (SPSS) (version 28). Descriptive statistics, including percentage, mean score, and frequency, were used to analyze quantitative data related to the impact course.

### 2.2. The Qualitative Component

#### 2.2.1. Qualitative Data Collection and Analysis

Students from three cohorts (2022–2025) were asked to submit a reflective essay as part of their coursework and professional training as critically reflective nurses [44,45]. In total, 102 students submitted essays, representing 54.5% of all course graduates across five cohorts. The act of reflection enables the nursing student to process and understand their own experiences in a given situation, learn from their experiences, and improve their practice [46]. This essay was started at the beginning of the course and was meant to reflect the learning process throughout the semester. The reflective essay was collected at the end of the course. To assist in the reflective process, 31 guiding questions were provided across four categories:

1. The student’s prior experience before the course. For example: Before the course, to what extent and in what ways were you exposed to entrepreneurship, and impact entrepreneurship in particular? Before starting the course, what was your attitude toward entrepreneurship, and nursing entrepreneurship specifically?
2. The student’s perspectives. For example: Following the course, what is your attitude toward entrepreneurship, and nursing entrepreneurship in particular? Has your attitude changed as a result of the course? If so, how? How do you perceive the relationship between impact entrepreneurship and nursing?
3. The student’s project in the course. For example: Why did you choose your project topic? What aspects of preparing the project did you enjoy and what aspects did you not enjoy, and why? What did you learn from the project development process?
4. The student’s learning process. For example: Were and how were the classroom topics relevant to preparing your project? In your opinion, to what extent and in what ways does the course contribute to entrepreneurial action in nursing?

We developed the guiding questions based on findings from previous studies regarding the effects of entrepreneurship and innovation on nurses and nursing students. A pilot was conducted, after which some questions were clarified, modified, removed, and added.

Students were not required to address any or all of the questions. Students were also able to address additional aspects that may not have been covered in the guiding questions. Responses to the questions could be structured or presented in an open narrative style, based on individual preference.

Thematic analysis was then utilized to inductively identify significant themes in the reflective essays [47]. Students were given pseudonyms for the reflective essays to maintain confidentiality. Two researchers independently analyzed the reflective essays to identify themes. First, the researchers conducted a holistic reading of the essays. This was followed by an open coding stage, where the data was categorized into different themes. Later, in an axial coding, these themes were refined and several similar themes were combined to provide more meaningful themes. Most themes that were identified by the researchers were similar, and those that were not, were discussed, revised, and refined through iterative discussion.

#### 2.2.2. Trustworthiness

Four aspects of trustworthiness were ensured during the qualitative study. Credibility, or the degree to which the research data actually measure what they intend to measure, was ensured in several ways. These included the utilization of a well-established research method, thematic analysis; triangulation, whereby both qualitative and quantitative methods were used; ensuring honesty, by allowing the students to express themselves freely in the essays; and frequent debriefing sessions by the researchers. The transferability of these data is made clear through the boundaries of the study population, specifically who was included in this study, how and when data were collected. The dependability of the study is highlighted in the detailed study methods, as well as the utilization of two study methods. Finally, confirmability was guaranteed by providing a step-by-step explanation as to how the data were obtained and later analyzed [48].

### 2.3. Ethical Considerations

Ethics approval was obtained from the Jerusalem College of Technology Ethics Committee (reference number 001_22). Questionnaires and reflective essays were de-identified and did not collect participants’ identifying personal information. The first part of the first section of the questionnaire asked the participant for their permission to participate in the research. By replying positively to that question, the participants gave their consent for participation. Students were also asked to provide informed consent for the use of their essays; essays from students who did not consent were excluded. The essays were anonymized, and the students’ names used in this article are pseudonyms.

## 3. Results

### 3.1. Quantitative Data Results

All respondents were women participating in the MSN-NP program in either the geriatric or the palliative care tracks and already hold a bachelor’s degree in nursing. Most of the respondents were over the age of 40 (58.7%) and married (91.3%). Over 43% worked in geriatrics or internal medicine; among the remaining participants, the work setting was split evenly between surgery and intensive care and community. Over 30% of nurses reported having worked in nursing between 25 and 30 years, while over 52% reported working in nursing between one and fifteen years (Table 1).

Out of the 46 respondents, only 5 (10.9%) reported that they had prior experience in entrepreneurship and the impact field. The majority (89.1%) of the respondents had no previous experience. Most of the participants (82.6%) stated that they leveraged their personal work experience when deciding on a theme for their impact project in the course. This group seems to have been significantly affected by their patient experiences, using those experiences to guide their plans and strategies. Contrastingly, the majority (54.3%) reported that their personal experiences with the healthcare system as a patient or relative of patients did not contribute to the selection of their topic. They may have chosen the topic based on a range of other factors, such as their professional knowledge and concern about societal issues (Table 2).

The participants were also asked several questions pertaining to their assessment of impact entrepreneurship and their own impact project. When asked if entrepreneurship might culminate in a personal job change, on a scale of 1 (not at all) to 6 (yes, absolutely), only two participants (4.3%) agreed that their entrepreneurship can certainly lead to a job change, and 11 participants (23.9%) responded that their entrepreneurship would certainly not lead to a job change. Most participants (56.5%) chose options 3–5 on the scale, suggesting that many students would not rule out a job change related to entrepreneurship. Furthermore, two thirds (67.4%) of the students thought that their impact project was feasible, while only 13% thought that it was not feasible. Over half (54.3%) have already undertaken activities to promote the execution of their project. Given that the realization of the proposed project was not a formal requirement of the course, this finding indicates that most students were committed to their impact entrepreneurship and project even beyond the course. Finally, regarding the existence of gender gaps in entrepreneurship and innovation, the majority (62.2%) stated that they believed gender gaps exist (Table 3).

### 3.2. Qualitative Data Results

Seven themes emerged that describe the experiences, perceptions, and feelings of the students regarding entrepreneurship and the course:

(1) Participants’ feelings prior to the course.
(2) Change in perspective towards entrepreneurship.
(3) Nurses as entrepreneurs.
(4) The course’s contribution to students’ personal and professional development.
(5) The added value of experiential learning.
(6) Gender and entrepreneurship.
(7) Nurses’ earning potential.

#### 3.2.1. Participants’ Feelings Prior to the Course

Many students described how they felt when they registered for the course and when they read the syllabus. Most students described feeling wary about the course, not quite understanding how it fits in with the curriculum. Before the course, participants reported that they did not understand the relationship between nursing and entrepreneurship.

*I was stressed when I first saw the course requirements; I felt it was very abstract to me. (Rivka)*

*When I first saw the instructions for the final project, I freaked out. This is not my strong side. I had no experience in the field of entrepreneurship and was not exposed to this field at all. (Lea)*

Furthermore, students indicated that they were unfamiliar with the course topics, including economics and business.

*I have no connection to topics like economics, finance, and business. … For this project, we needed to think about the economic and business side and connect ideas with action. This was a challenge for me but was what made the project special. (Rivka)*

Some students expressed excitement about the idea of delving into the field of entrepreneurship. They explained that, while they held an intuitive interest in the topic, they had not previously had the opportunity to engage with it.

*I am entrepreneurial by nature. Ever since I can remember, crazy ideas have been running through my head, and I enjoy turning them into reality. … In the past, I thought of several medical inventions. For one, I even submitted a research protocol. (Nava)*

*Entrepreneurship always interested me, but I had no experience with it. (Tzippora)*

#### 3.2.2. Change in Perspective Towards Entrepreneurship

Once the students began to understand the field of entrepreneurship and specifically impact entrepreneurship, they discussed their appreciation of entrepreneurship, and how it contributes to society. Many students indicated that the course changed the way they viewed entrepreneurship and provided them with the tools to become entrepreneurs in their own right.

*The course changed the way I see entrepreneurship. I used to think it was only relevant for high-tech … but I learned it can be applied everywhere, by any person who has an idea in their field. (Gali)*

*Through my project, I was exposed to the field of impact entrepreneurship, and I found myself connecting much more and better understanding the benefits and the need of impact entrepreneurship. (Rivka)*

Students reported that exposure to systematic models and concrete steps in entrepreneurship rendered this field more accessible to them.

*My perspective changed a bit after the course because I understood that entrepreneurship is not some vague thing that has no rules. There are many steps that really focus the field of impact entrepreneurship in nursing and make entrepreneurship something that is clearly defined. From the beginning, you can examine it using appropriate models to see if your idea will pay off. (Elana)*

Furthermore, many students realized that impact entrepreneurship does not involve a conflict of values. They discovered that entrepreneurship and social contribution complement each other, allowing impact entrepreneurs to innovate in ways that benefit both themselves and society.

*I don’t see any tension between entrepreneurship and contribution to society. To the contrary. I suppose that there are people who say that there is a conflict, because contribution to society goes with a mission, while entrepreneurship goes with money. … But we can have entrepreneurship with money and a mission and contribution to society. These four elements go together well. (Michelle)*

Other students stated that this course only reaffirmed their existing perspectives on entrepreneurship in nursing.

*Truthfully, it didn’t change my approach because the course discussed topics that I’ve had in my head ever since I’ve known my adult self. (Nava)*

*My views didn’t change because of the course. The opposite, in fact. Medicine and nursing go hand in hand with entrepreneurship. It’s no coincidence that there are numerous startups in the field of biotechnology that are working on providing solutions to problems in the medical world. (Batya)*

These reflections indicate that the course was less transformative for students who entered with a prior interest in entrepreneurship.

#### 3.2.3. Nurses as Entrepreneurs

Students also expressed their thoughts on nurses as entrepreneurs and innovators. Many described how nurses might not be traditionally associated with entrepreneurship, but that throughout the course, they realized that not only can nurses be innovators, but that, given their closeness with the patient population, they should be innovators.

*Today, I am more motivated and believe in our ability, as nurses, to influence, initiate, implement changes, and earn much more than the average nurse’s salary. I understand that we are capable of identifying difficulties, mapping barriers, offering creative solutions, and performing every step from A to Z to make the change. (Michelle)*

*Ideas that come from the field are better and more successful. (Leora)*

*We, as nurses, can better understand the unmet needs of the patients, and … can dream up different situations or devices that can promote our patients’ welfare. As a nurse, … the ability to give to my patients and improve their quality of life, even a little bit, is of utmost importance. (Elana)*

Students made a connection between the nurses’ role and responsibility of social advocacy, e.g., identifying unaddressed needs and mapping structural barriers, and their capacity to find and promote systemic solutions to these problems through impact entrepreneurship. Participants also emphasized that as nurses, the course was their first opportunity to learn this subject, suggesting that this subject is clearly underdeveloped in nursing.

*This course promotes entrepreneurship in nursing by exposing nurses to a whole other world that I think I personally would not have been exposed to anywhere else. (Gali)*

#### 3.2.4. The Course’s Contribution to Students’ Personal and Professional Development

Students described how the course contributed to their personal and professional growth. Professionally, the course enabled them to look at aspects of the healthcare system differently. It also gave them more confidence to self-develop and challenge themselves within their profession, helped them expand their horizons, and made them feel empowered.

*The project contributed to my professional life in that I was exposed to unique ideas that my classmates presented and to new concepts in the world of nursing and rehabilitation. (Atara)*

*The project I worked on is very dear to my heart. I learned about mindfulness, and I was excited to work on the project and integrate it into my everyday life and into my professional life. My project integrates really well with my nursing work and enables me to better help and care for my patients in the clinic. (Lea)*

Personally, the course changed the way the students saw themselves and their colleagues. They expressed how they learned about their abilities, that they are or can be entrepreneurs. The course provided them with tools to be active in changing the reality in the field.

*The course certainly contributed to changing my way of thinking. … Because of this, I have more confidence in the field of nursing, in nurses, in myself, in my potential, and in my colleagues’ potential to go far and achieve any professional dream we choose. (Michelle)*

*I learned about myself that the topic of entrepreneurship is not new to me, and I may even enjoy dipping my toes into it in the future. (Rivka)*

This and other quotes demonstrate that the course helped students to turn implicit knowledge into explicit knowledge. Moreover, students observed that, while nursing is often regarded as an altruistic rather than self-centered profession, this does not preclude them and other nurses from being creative and developing their own ideas.

*I think it’s a great idea to include entrepreneurship in nursing school. We hear so much about how nursing is a calling. This course feels like a way to balance this and say to nurses and nursing students: “you may have chosen an altruistic profession, but you can also learn how to maneuver yourself in this wonderful profession, find yourself a niche, or—if one doesn’t exist—carve one out especially, because you can.” (Michelle)*

However, some students felt that entrepreneurship studies did not need to be taught to every nurse, but rather only to those who considered it relevant to their future.

*I think that nursing students should be able to choose if they want to study entrepreneurship; it’s not for everyone. Some people prefer not to take initiative and a course like this could make them feel threatened in a way. I know some students were nervous about the course. (Elana)*

#### 3.2.5. The Added Value of Experiential Learning

Over the course of the semester, the students had two educational tours and guest lecturers with various experiences in impact entrepreneurship. One tour was usually at Zichron Menachem, an Israeli non-profit organization that supports children with cancer and their families. There, the students got to see first-hand innovative devices and programs that were developed to assist the patients. The other tour has evolved over the years—for example, a visit to a multicultural neighborhood in Jerusalem, where students learned about different communities that make up the patient population, each with unique needs and creative community-based solutions and initiatives. One theme that repeated itself in nearly every reflective essay was the effect that these tours and lectures had on the students and their perspective on entrepreneurship and innovation.

*The course included both theoretical material and two tours that really contributed to me and led me to open other ways of thinking. The wonderful venture of Zichron Menachem really impressed me and gave me hope that we can develop frameworks of holistic support and treatment in the community and provide a solution for vulnerable populations. (Tzippora)*

*I was so charmed by the visit to Zichron Menachem. I was in awe of the idea and its execution, particularly by Menachem’s parents, the founders. It’s an inspirational place. Additionally, hearing from Avivit, who is a graduate of the Jerusalem College of Technology and studied what I am currently studying, gave me a concrete channel to what I should expect of myself, kind of a role model. (Liat)*

Liat and other students emphasized the meaningful learning from graduates of this course, who succeeded in their entrepreneurship and innovative projects and can become role models for the current students.

#### 3.2.6. Gender and Entrepreneurship

Several students expressed their thoughts about how women are not often thought of as entrepreneurs. Primarily, these students wrote that they believe this is a misconception, similar to the idea that women cannot do certain jobs because they must take care of their children. Instead, women can and should be leading entrepreneurs and have the skills to do so.

*I deny the existence of a relationship between gender and entrepreneurship and business. (Nava)*

*The relationship between gender and entrepreneurship and business automatically makes me think of the idea “women are less successful because they are at home with the children.” … I strongly disagree with these claims; I believe that women can succeed in entrepreneurship and business just like men. They can succeed even more because they have creative thinking skills, the ability to multitask, and resourcefulness. (Michelle)*

Furthermore, the students stated that they believed that anyone who wants to try to succeed has the power to do so, regardless of their gender.

*Anyone who wants to succeed can try and succeed. I don’t see a reason to delay anything because of labels. (Nava)*

#### 3.2.7. Nurses’ Earning Potential

The students discussed nurses’ earning potential, particularly to what extent their income is limited within the profession. Several noted that nurses’ salaries are notoriously low. However, many people enter nursing because they view it as a calling, rather than as a way to make money.

*I can’t see myself working in a different field. Even when this field wears me out, I can’t imagine myself in any other place. Nursing is my calling. (Atara)*

Meanwhile, students felt that the idea that nurses have limited earning potential is wrong, or at least partially wrong.

*I do not agree with the claim that whoever chooses to go into nursing gives up the possibility to make money. (Eleanor)*

*There are ways to earn a higher salary. … You can work in several places or in a hi-tech company that has nursing position with better salaries. (Atara)*

Importantly, most students agreed that incorporating entrepreneurship into their nursing careers could increase earning potential. It offers nurses an opportunity to supplement their income, which in turn may encourage more people to enter the profession and attract highly qualified individuals.

*The course showed me that not every person who goes into nursing gives up their earning potential. (Elana)*

*I believe that thinking big, ignoring background noise and negative thoughts, and believing in action—will make it possible for nurses to go far. (Eleanor)*

*If there is a way to pay them and encourage action, there is nothing better than that. Money is not a bad word, especially if it is for a good cause. As soon as money is poured into the profession, more and more quality people will join this circle of action and this will promote competition for a good cause. (Gali)*

Overall, these reflections illustrate an awareness among the students of the interconnection between professional fulfillment, financial independence, and social contribution. Following the course, the participants challenged traditional perceptions of nursing as solely altruistic work, instead portraying it as a dynamic, aspirational profession that can offer personal growth, increased earning potential, and meaningful societal impact.

## 4. Discussion

This study aimed to assess nurse practitioner students’ experiences and perceptions related to an impact entrepreneurship course. In their reflective essays, many students highlighted how this course empowers nurses to be innovators, how it may possibly increase their earning potential, and how they can make a difference for their patients. Many students also shared how this course contributed to their professional and personal development.

For many students, this course was their first exposure to economic and financial aspects and theories, and how these can be applied to the field of healthcare [20]. The students were taught to think in terms of profit and financial loss, which, as reported by many, had been a foreign concept to them [14]. This was mainly because nursing is considered an altruistic profession, and this concept of altruism is at the core of many nursing courses. Students also stated that they were stressed about the requirements of the course because they had no background in business or economics. However, the results of both the survey and the reflective essays indicate that most students grew more comfortable with the topic and requirements, and many groups of students have continued building and implementing their projects even after the course itself ended.

The survey findings indicate that most students did not see entrepreneurship as a career change. However, over 32% reported leaning toward entrepreneurship (scoring 4 and 5 on this scale), suggesting that this group may be more inclined to integrate entrepreneurial practices within their current roles (intrapreneurship). This demonstrates that while most nursing students do not plan to pivot into innovation fully, participation in this course can encourage them to become involved in related enterprises [27]. This quantitative result complements the qualitative finding that incorporating entrepreneurship into nursing careers could help supplement and increase income. Taken together, the survey and essay findings suggest that adding an entrepreneurial dimension to existing nursing work is both realistic and may yield economic benefits for nurses. This, in turn, may enhance the profession’s prestige and attract more individuals to pursue nursing as a career.

In their essays, many students explained that following the course, they clearly see the connection between their nursing practice and entrepreneurship. In the survey, most students (82.6%) reported that their work experience influenced their projects. This is unsurprising, given that the calls for nurses to become more involved in entrepreneurship stem from their unique perspective of the patient experience and the needs of the healthcare system [20,22]. The students in the NP program have vast professional experience in caring for older adults and patients receiving palliative care, and a deep understanding of the various patient needs and systemic barriers. These nurses also have the tools to evaluate alternative solutions for structural problems [49,50,51]. Their professional experience is a resource of great importance to the professional level of the projects they proposed and their ability to implement them. Indeed, two-thirds of respondents believed their course project was feasible. Course graduates will be able to deliver meaningful and impactful care through a deep understanding of their field and newly acquired knowledge of social enterprise [17].

Kusumawijaya [52], Maharani et al. [53], Saif and Ghania [54], and Akhtar et al. [55] recognized that innovation attitude, locus of control, and self-efficacy facilitate women’s motivation for success. Participating in an impact entrepreneurship course and receiving tools that assist with innovation, business administration, and the development of a business network likely have a positive effect on nurses’ self-efficacy [20]. Engaging in entrepreneurship and innovation can lead to a change in thinking from “passive” thinking, which tends to characterize many in the nursing profession, to a more proactive and egalitarian thinking. Accordingly, the students reported gaining a sense of empowerment, which is particularly significant for female students [22,56] and for those working in professions historically constructed as “feminine,” such as nursing [57].

The majority of students (62.2%) stated that there is a gender gap in entrepreneurship and innovation, while only 15.6% believed there was no such gap. The reflective essays shed additional light on this perception. Students explained that the notion that women are less entrepreneurial than men stems from sexist views, emphasizing instead the valuable skills many women possess, such as multitasking [58,59]. This is in line with many studies, both in the world of healthcare and outside of it [60,61,62]. Despite gains in recent years to encourage more women to take part in innovation and entrepreneurship in a variety of fields, including healthcare, science, technology, engineering, and mathematics, there are still gaps, and needs of women are not always considered in relation to innovation [63]. In the world of health, this is manifested by an overrepresentation of men in clinical trials, unaddressed physiological differences, and an overreliance on male health outcomes to drive development [64]. Providing female nurses with the tools to be innovators and entrepreneurs in healthcare will not only serve to bridge this gender gap, but will also provide a louder female voice to issues that impact entrepreneurship may address, in this case, for older females and female patients with terminal diseases [63].

Moreover, Israel is experiencing increasing rates of aging [34]. This requires special resources for caring for the older population. However, the relative share of the gross domestic product invested in healthcare in Israel is particularly low, further highlighting the need for efficient and suitable solutions at a relatively low cost [65]. Additionally, there is a mistaken belief that older adults are technology-averse and are reluctant adapters of new technologies. Though it has been debunked in research, entrepreneurs are still more hesitant to develop gerontechnology [38]. This leaves a gap in the market that could potentially be filled by experienced geriatric nurses. The nurses who participated in this course expressed interest in engaging in such entrepreneurship in the future. This course provides a platform for raising new ideas and finding viable solutions for the healthcare system and may help identify tools to reduce social disparities in health.

Finally, despite the course’s considerable benefits, the findings highlight a dilemma regarding its status as a mandatory course. Some students argued that, due to its unconventional nature and distinctive place within the traditional curriculum, it would be more suitable as an elective. On the other hand, students who were initially uninterested in the subject—and who likely would not have enrolled in such an elective—reported experiencing a significant transformation as a result of the course, which opened a new window of opportunity for them. Future programs should carefully balance between allowing choice and ensuring exposure to transformative learning experiences that challenge students’ assumptions and broaden their horizons.

### Limitations

There are several limitations to this study. First, the survey response rate was relatively low, and it is possible that those who responded either enjoyed the course or felt they had specific feedback to share [66]. This also resulted in a relatively small sample size. Additionally, all participants were women, and a significant proportion were religious, which could introduce selection bias. The sample’s homogeneity may have influenced the findings and limits the study’s generalizability. Moreover, we examined our own educational program and students, which may introduce bias despite the measures taken to minimize this risk. Finally, this NP program is currently the only one in Israel offering this track, and we were unable to compare syllabi or assess outcomes in other graduate nursing programs, limiting the generalizability of our findings. More educational programs and research are needed on this underdeveloped and under-researched subject. Future studies examining impact entrepreneurship programs across different countries and diverse educational settings, including various undergraduate and graduate nursing programs, could increase sample diversity and size, thereby enhancing the generalizability of the findings.

## 5. Conclusions

This article showed how nurse practitioner students engaged with and made sense of an impact entrepreneurship course, addressing a significant gap in understanding how impact entrepreneurship programs shape learners’ perceptions, competencies, and professional practices. By highlighting a program rarely implemented for nurses, this article provides new insights into preparing nursing students for entrepreneurial roles and helps bridge the gap between nursing education and the rapidly developing field of impact entrepreneurship.

This research suggests that an impact entrepreneurship course for nurses can serve as a tool for empowerment. Exposure to impact entrepreneurship can help nurses identify creative solutions to challenges in their work and contribute to cost savings in the healthcare system. An impact entrepreneurship course provides significant benefits to students and is likely to benefit their patients as well as the broader population. This is the first course of its kind in an NP track, and we found that, following the course, students felt better prepared for success in today’s innovation-driven economy. We recommend that professional and educational institutions worldwide incorporate the emerging framework of impact entrepreneurship into nursing curricula and practice, encouraging nurses to take a leadership role in this field within the healthcare system and beyond. Further research is needed to identify the dynamic changes in the nursing profession and education that can contribute to innovation and entrepreneurship.

## Figures and Tables

**Table 1:** Sociodemographic and occupational characteristics, *n* = 46.

	Categories	N (%)
Age	20–40	19 (41.3%)
	40+	27 (58.7%)
Marital status	Not married	4 (8.7%)
	Married	42 (91.3%)
Religious observance	Secular	18 (40%)
	Religious	6 (13.3%)
	Very religious (ultra-Orthodox)	21 (46.7%)
Work setting	Geriatrics and internal medicine	20 (43.5%)
	Surgery and intensive care	13 (28.3%)
	Community	13 (28.3%)
Level of nursing education	Bachelor’s	24 (53.3%)
	Master’s	21 (46.7%)
Nursing experience (in years)	1–10	12 (26.1%)
	10–15	12 (26.1%)
	15–20	8 (17.4%)
	25–30	14 (30.4%)

**Table 2:** Personal perspectives on impact entrepreneurship, *n* = 46.

Personal Perspectives	Yes
Do you have previous experience in entrepreneurship and the field of impact?	5 (10.9%)
Was your impact project based on your personal experience at work?	38 (82.6%)
Was your impact project based on your personal experience as a patient or relative of patients in the healthcare system?	21 (45.7%)

**Table 3:** Students’ assessment of impact entrepreneurship and their impact project, *n* = 46.

	Categories	N (%)
Can entrepreneurship be a job change for you? (1 = not at all; 6 = yes, absolutely)	1	11 (23.9%)
	2	7 (15.2%)
	3	11 (23.9%)
	4	8 (17.4%)
	5	7 (15.2%)
	6	2 (4.3%)
Do you believe the project you proposed is feasible?	Yes	31 (67.4%)
	No	6 (13%)
	Maybe, but not now	9 (19.6%)
Have you undertaken any activities to promote the execution of the project you proposed?	Yes	25 (54.3%)
	No	21 (45.7%)
Do you think there are gender gaps in entrepreneurship and innovation?	Yes	28 (62.2%)
	No	7 (15.6%)
	I’m not sure	10 (22.2%)

## Data Availability

The survey data presented in this study are available on request from the corresponding author. The qualitative data are available but not shared. The reflective essays were written by students who provided consent for their essays to be used only by the researchers.

## Public Involvement Statement

No public involvement in any aspect of this research.
